# Enhancement of Endometrial Receptivity by *Cnidium officinale* through Expressing LIF and Integrins

**DOI:** 10.1155/2019/7560631

**Published:** 2019-11-16

**Authors:** Tae-Wook Chung, Mi-Ju Park, Hoyoung Lee, Keuk-Jun Kim, Cheorl-Ho Kim, Hee-Jung Choi, Ki-Tae Ha

**Affiliations:** ^1^Department of Korean Medical Science, School of Korean Medicine and Healthy Aging Korean Medical Research Center, Pusan National University, Yangsan, Gyeongnam, Republic of Korea; ^2^Clinical Medicine Division, Korea Institute of Oriental Medicine, Yuseong-gu, Daejeon, Republic of Korea; ^3^Department of Clinical Pathology, DaeKyeung University, Gyeongsan, Gyeongbuk, Republic of Korea; ^4^Department of Biological Science, Sungkyunkwan University, Suwon, Kyunggi-do, Republic of Korea

## Abstract

Improvement of endometrial receptivity is necessary for successful embryo implantation, and its impairment is associated with female infertility. In this study, we investigated the effect of the roots of *Cnidium officinale* Makino (CoM) on endometrial receptivity in both in vitro and in vivo model of embryo implantation. We found that CoM enhanced the adhesion of JAr cells to Ishikawa cells by stimulating expression of leukemia inhibitory factor (LIF) and integrins. In addition, blocking of LIFR using hLA or neutralization of integrins *α*V, *β*3, and *β*5 using antibodies significantly reduced the enhanced adhesion between JAr cell and CoM-treated Ishikawa cells, indicating that LIF and integrin play an important role in trophoblast-endometrium adhesion for embryo implantation. Furthermore, we identified that CoM significantly improved the implantation rate of blastocysts in the mouse model of RU-induced implantation failure. By collecting these results, here, we suggest that CoM has a therapeutic potential against female infertility associated with decreased endometrial receptivity.

## 1. Introduction

Embryonic implantation for a successful pregnancy is indispensable, which involves a complex process that requires molecular interactions of the endometrium and blastocyst [1]. During the window of implantation, the optimal environment of endometrium for adhering the embryo implies ultimately enhanced endometrial receptivity. This also affects embryo development and placenta formation [2]. The receptive endometrium is regulated by the interaction between hormones, growth factors, and cytokines [3]. Among cytokines in the endometrium, leukemia inhibitory factor (LIF) plays an important role in embryo implantation and is attributed to endometrial receptivity. LIF binds with its receptor (LIFR) and the coreceptor glycoprotein 130 (gp 130), resulting in activation of downstream signaling through signal transducer and activator of transcription factor 3 (STAT3) [4, 5]. Interestingly, the expression of STAT3 is only possible during a certain period of endometrial receptivity [6]. Among the adhesion molecules that are regulated by cytokines such as secreted LIF, integrins are essential proteins to establish the required physical interaction between endometrium and blastocyst at the time of implantation. Like a LIF, the expression of integrins is used as a marker of endometrial receptivity [7, 8].

The extract of *Cnidium officinale* Makino (CoM), which belongs to the Umbelliferae family, has been used as traditional herbal medicines to treat pain, inflammation, and menstrual disturbance in Asia [9]. Recent studies demonstrated that CoM has several pharmacological activities, such as anti-inflammation, antitumor, antimetastasis, and antiangiogenesis [9–13]. In a field of female disorders, several papers demonstrated that herbal formulae including *C. officinale* showed the estrogenic potential and antileukorrhea effect [14, 15]. However, there are still no previous studies reporting the effect of CoM on the enhancement of the endometrial receptivity.

In this study, we showed a potential effect of CoM on the enhancement of the endometrial receptivity. The molecular regulation underlying CoM-increased enhanced endometrial receptivity was related to the expression of LIF and integrin molecules. From these results, we suggest that CoM might be a good candidate for a novel drug for ameliorating female infertility related to decreased endometrial receptivity.

## 2. Methods

### 2.1. Materials

Anti-LIF was supplied by Santa Cruz Biotechnology (Santa Cruz, CA). Anti-integrin *α*V, anti-integrin *β*3, and anti-integrin *β*5 were purchased from Abcam (Cambridge, UK). A soluble antagonist for human LIFR (hLA) was purified in our laboratory, as described previously [16]. Mifepristone (antagonist for progesterone receptor; RU486) and human chorionic gonadotropin (hCG) were obtained from Sigma-Aldrich (St. Louis, MO). Gonadotropin from pregnant mare serum (PMSG) was purchased from LEE BioSolutions (Maryland Heights, MO).

### 2.2. Preparation of Herbal Extract

**Figure 1:**
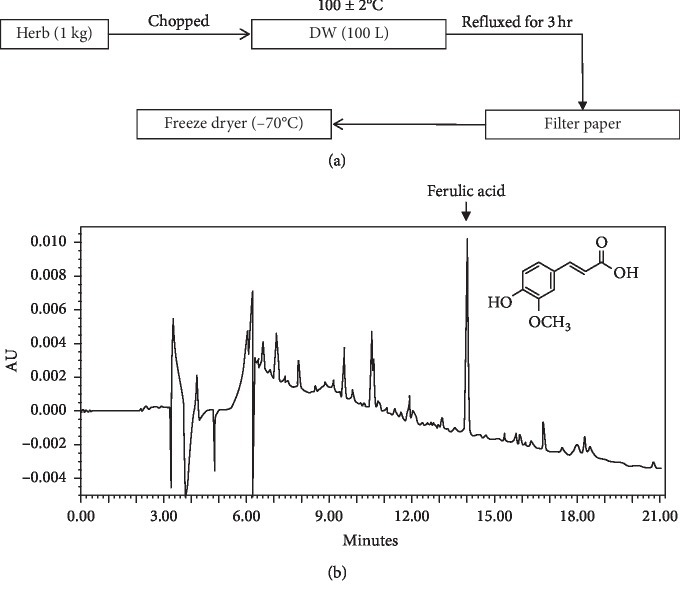
Method for water extraction and fingerprinting of CoM. (a) Schematic presentation of water extraction process of CoM. (b) The water-extracted CoM was analyzed by HPLC.

Dried roots of *C. officinale* were purchased from Omni Herb (Daegu, Korea) in 2012. This material was confirmed by Dr. Goya Choi of Korea Institute of Oriental Medicine (KIOM) (Daejeon, Korea). A voucher specimen (2-17-36) was deposited at the Clinical Medicine Division, KIOM. The extraction method is shown in Figure 1(a). Briefly, the extract was prepared in KIOM's laboratory from a mixture of chopped crude herbs that was extracted using distilled water at 100 ± 2°C for 3 h by reflux extraction (COSMOS, Kyungseo Machine Co., Incheon, Korea). The solution was filtered through filter paper. The filtered solution was concentrated using a vacuum evaporator (Ev-1020; Daihan Scientific Co., Ltd, Gangwon-do, Korea). The extract was freeze-dried to create a powder (Genesis 25L; SP Scientific, Stone Ridge, NY). The prepared powder (CoM) was stored at −70°C.

### 2.3. High-Performance Liquid Chromatography (HPLC) Analysis

The lyophilized extract (10 mg) was dissolved in 70% methanol (5 mL) and then filtered through a 0.2 *μ*m membrane filter (Woongki Science Co., Ltd., Seoul, Korea) before being injected into HPLC for component analysis. The ferulic acid (Sigma-Aldrich; purity over 98.0%) was used as a standard indicator of CoM in this study [17]. The HPLC-grade solvents, methanol, acetonitrile, and water were obtained from J. T. Baker (Phillipsburg, NJ). Trifluoroacetic acid (analytical reagent grade) and the standards were procured from Sigma-Aldrich. The HPLC system consisted of a Waters Alliance 2695 system coupled with a 2998 photodiode array detector (Waters Co., Milford, MA). Data processing was performed with Empower software (version 3; Waters Co.). The ferulic acid in CoM was separated using a Luna 5 *μ*m C_18_ 100 A column (4.6 × 250 mm, 5 *μ*m particle size; Phenomenex Inc., Torrance, CA). The monitoring was performed at 240 nm for ferulic acid. The mobile phases consisted of water with 0.1% (v/v) distilled water (solvent A) and trifluoroacetic acid (solvent B) at a flow rate of 1.0 mL/min. The gradient conditions changed as presented in Table 1. The injection volume was 10 *μ*L.

### 2.4. Cell Culture

The endometrial Ishikawa cell line was provided by Dr. Jacques Simard (CHUL Research center, Quebec, Canada) and cultured with Dulbecco's modified Eagle medium (DMEM; Welgene, Daegu, Korea) containing 10% heat-inactivated fetal bovine serum (FBS; Sigma-Aldrich) and 1% penicillin/streptomycin (PSA; Thermo Fisher Scientific, Waltham, MA). The choriocarcinoma JAr cells were obtained from Korean Cell Line Bank (Seoul, Korea) and cultured with Roswell Park Memorial Institute 1640 (RPMI1640; Welgene) containing 10% FBS and 1% PSA. These cells were maintained at 37°C in an atmosphere containing 5% CO_2_/air.

### 2.5. Cell Viability Assay

The cells were cultured in 24-well plates with indicated concentrations of CoM for 24 h. Then, the media were replaced with 3-(4,5-dimethylthiazol-2yl-2,5-diphenyltetrazolium bromide (MTT; Sigma-Aldrich)) solution (0.5 mg/mL) and incubated in 37°C for 4 h. The formed formazan crystals were fused with dimethylsulfoxide/ethanol (v/v, 1 : 1) solution. The cell viability was estimated by measuring the absorbance at 540 nm with a microplate reader (SpectraMax M2; Molecular Devices, San Jose, CA).

### 2.6. Adhesion Assay

Adhesion assay between JAr and Ishikawa cells were prepared as described previously [18]. To make cell monolayers, Ishikawa cells (1.5 × 10^6^ cells) were seeded into 6-well plates and incubated with CoM treatment for 48 h. The JAr cells (2 × 10^6^ cells) were labeled with 5-chloromethylfluorescein diacetate (CMFDA) fluorescence dye (CellTracker Green; Life Technologies, Carlsbad, CA) for 15 min. To check adhesion between Ishikawa and JAr cells, labeled JAr cells were added to the Ishikawa cell monolayer. After shaking gently for 30 min, the cells were washed to remove unbound JAr cells. The attached cells were visualized with a fluorescence microscope (Axio Imager M1; Zeiss, Oberkochen, Germany) and counted.

### 2.7. Quantitative Real-Time PCR (qRT-PCR)

Total RNA from each sample was isolated using RiboEx™ (GeneAll, Seoul, Korea), and cDNAs were synthesized with oligo-dT and M-MLV reverse transcriptase (Enzynomics, Daejeon, Korea). According to the manufacturer's instructions, qRT-PCR was performed with Qiagen Rotor-Gene Q Real-time PCR Detection System using QuantiNova™ SYBR Green PCR kit (Qiagen GmbH, Düsseldorf, Germany). The primers used in this study were as follows: *LIF*, forward 5′-GGCCCGGACACCCATAGACG-3′ and reverse 5′-CCACGCGCCATCCAGGTAAA-3′; *ITGAV*, forward 5′-ATGCTCCATGTAGATCACAAGAT-3′ and reverse 5′-TTCCCAAAGTCCTTGCTGCT-3′; *ITGB3*, forward 5′-CTGCCGTGACGAGATTGAGT-3′ and reverse 5′-TGCCCCGGTACGTGATATTG-3′; *ITGB5*, forward 5′-ACCTGGAACAACGGTGGAGA-3′ and reverse 5′-AAAAGATGCCGTGTCCCCAA-3′; and *β-actin*, forward 5′-CAAGAGATGGCCACGGCTGCT-3′ and reverse 5′-TCCTTCTGCATCCTGTCGGCA-3′.

### 2.8. Western Blot Analysis

Total protein from each sample was extracted using 1% NP-40 lysis buffer (Thermo Fisher Scientific), separated by SDS-PAGE, and electrotransferred to nitrocellulose membranes (0.45 *μ*m; Thermo Fisher Scientific). The membranes were incubated with 1X blocking solution and then with primary antibodies, respectively. After the reaction with appropriate HRP-conjugated secondary antibodies, the bands were visualized using the ECL chemiluminescence system (GE Healthcare, Chicago, IL).

### 2.9. Animals

Male and female C57BL/6 mice (7–8 weeks old, weight 20–22 g) were purchased from Orient Bio, Co. (Seongnam, Korea). They were bred in a specific pathogen-free facility with free access to water and a standard diet under a 12 h light/12 h dark cycle. All experimental procedures were approved by the Animal Research Ethics Committee at the Pusan University of Korea (no. PNU-2018-1943).

### 2.10. Animal Models and Treatment

The embryo implantation failure model was performed as described previously [19]. A schematic diagram of an in vivo experimental schedule is shown ([Supplementary-material supplementary-material-1]). Briefly, forty female mice were randomly divided into four groups: control (Con), RU486 (RU), CoM + RU, and CoM. Female mice of CoM + RU and CoM or Con and RU groups were dosed daily with CoM (67.75 mg/kg/day) or normal saline solution for 15 days, respectively. The doses of CoM were determined by daily intake criteria for humans (16 g intake for an average human 60 kg) in the clinic, which is about 67.75 mg/kg after considering the yield of CoM extraction (25.48%). Forty-eight hours before mating to synchronize the estrous cycle of female mice, 5 IU PMSG was pretreated and then 5 IU hCG was intraperitoneally injected. All female mice of groups were then mated with males (ratio 1 : 1). The next day, vaginal plug was observed and it was designated as day 1. To induce embryo implantation failure model, RU486 (4 mg/kg/day) or corn oil was orally administered to female mice of RU and CoM + RU or Con and CoM groups daily from day 4 to day 7. All mice were sacrificed the next day after separating from males, and both uterine horns were excised to examine the number of implantation sites. The number of implanted embryos on each uterine horn was counted.

### 2.11. Statistical Analysis

The statistical significance from results was performed by a Student's *t*-test or one-way analysis of variance with Tukey's post hoc test using GraphPad Prism (GraphPad Software, San Diego, CA) and expressed as the mean ± SEM. The minimum significance level was set at a *p* value of 0.05. All experiments were independently performed at least 3 times, except for animal study.

## 3. Results

### 3.1. CoM Increases the Adhesion of JAr Cells to Ishikawa Cells

**Figure 2:**
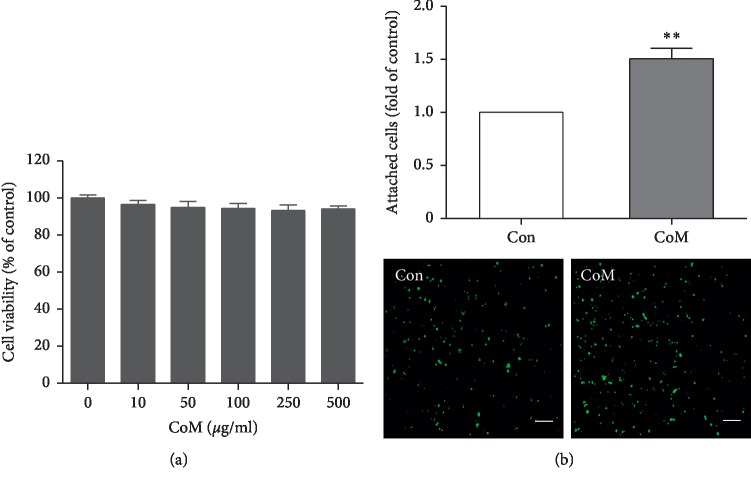
The effect of CoM on cytotoxicity and the adhesion of JAr cell to Ishikawa cells. (a) Ishikawa cells (1.5 × 10^6^ cells) were seeded and incubated with CoM at indicated concentration (up to 500 *μ*g/ml) for 24 h. The cell viability was measured by MTT assay. The data were expressed as percentage of controls and are shown as mean ± SEM of three independent experiments. (b) Ishikawa cells were treated with CoM (50 *μ*g/mL) for 48 h. CMFDA-labeled JAr cells were added onto the CoM-treated Ishikawa cells. The attached JAr cells were fixed, pictured using fluorescent microscopy, and manually counted. Data were calculated as mean ± SEM of three independent experiments. The data were expressed as fold of controls. ^*∗∗*^*p* < 0.01 for comparison between two groups (magnification ×50; scale bar = 5 *μ*m).

To evaluate the phytochemical property of CoM, the water-extracted CoM was analyzed by HPLC. The yield of CoM was 25.48% and the retention time of ferulic acid was 13.98 min on absorbance of 240 nm (Figure 1(b)). To evaluate the effect of CoM on the cell viability, MTT assay was performed. The results showed CoM did not induce significant cytotoxicity up to 500 *μ*g/ml (Figure 2(a)). In subsequent experiments, we used CoM concentration at 50 *μ*g/mL. To investigate the effect of CoM on embryo attachment for successful embryo implantation, we first performed an in vitro adhesion assay using human trophoblastic JAr cells and endometrial Ishikawa cells. As shown in Figure 2(b), CoM treatment elevated the cell adhesion between JAr cells and Ishikawa cells about 1.5 times compared with control.

### 3.2. The LIF and Integrins Mediate the CoM-Induced Endometrial Receptivity

**Figure 3:**
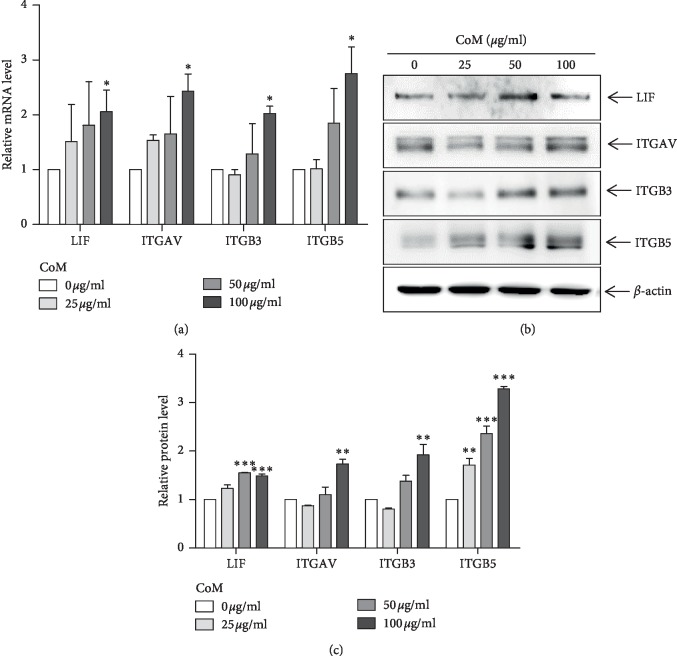
The enhanced expression of LIF and integrins by CoM in Ishikawa cells. Ishikawa cells were treated with indicated concentration of CoM for 24 h, and total RNA and protein were isolated. The expression levels of mRNA and protein for LIF, ITGAV, ITGB3, and ITGB5 were examined by qRT-PCR (a) and western blot (b), respectively. *β*-actin was used as an internal control. Band intensity was calculated by fold of control and is shown as mean ± SEM. ^*∗*^*p* < 0.05, ^*∗∗*^*p* < 0.01, and ^*∗∗∗*^*p* < 0.001 in comparison with control groups.

**Figure 4:**
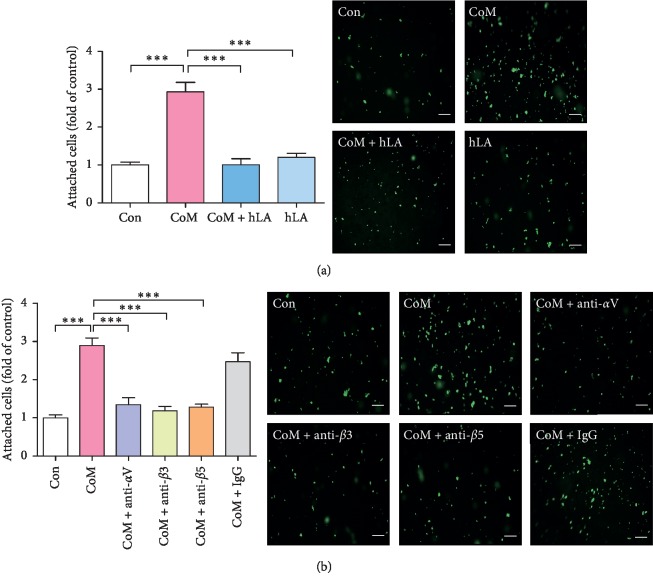
Effect of antagonist for LIFR and neutralization for integrins *α*V, *β*3, and *β*5 on the adhesion of JAr cells to CoM-stimulated Ishikawa cells. (a) Ishikawa cells were treated with or without LIF antagonist (hLA) (50 ng/mL) for 1 h and then CoM (50 *μ*g/ml) was added to hLA-pretreated Ishikawa cells for 48 h. (b) Ishikawa cells (1.5 × 10^6^ cells) were cultured in 6-well plates and treated with or without CoM (50 *μ*g/ml) for 48 h, and the cells were then incubated in the presence of integrin *α*V, *β*3, *β*5, and IgG antibodies for 2 h. CMFDA-labeled JAr cells were added onto Ishikawa cell monolayer. The attached cells were fixed and pictured using fluorescent microscopy. Data were calculated as mean ± SEM of three independent experiments and expressed as fold of controls. ^*∗∗∗*^*p* < 0.001 compared to each group (magnification ×50; scale bar = 5 *μ*m).

To determine the effect of CoM on the expression of LIF and integrins for endometrial receptivity, qRT-PCR analysis was performed using CoM-treated Ishikawa cells. As shown in Figure 3(a), CoM treatment elevated the mRNA level of *LIF*, *ITGAV*, *ITGB3*, and *ITGB5* in Ishikawa cells in a concentration-dependent manner. In addition, western blot analysis also showed that the expression of *LIF*, *ITGAV*, *ITGB3*, and *ITGB5* was enhanced by CoM stimulation (Figure 3(b)). To further investigate LIF-dependent adhesion between trophoblast and endometrium, we performed the adhesion assay on Ishikawa cells by hLA pretreatment. As shown in Figure 4(a), CoM increased the adhesion of JAr cells to Ishikawa cells, whereas pretreatment with hLA markedly alleviated the adhesion by inhibition of the LIF/LIFR signaling pathway. Similarly, we next investigated whether integrins play a role in CoM-enhanced endometrial receptivity toward trophoblast. The treatment of integrin *α*V, *β*3, and *β*5 antibodies for neutralization significantly reduced the enhanced adhesion between JAr cell and CoM-treated Ishikawa cells (Figure 4(b)).

### 3.3. CoM Improves In Vivo Embryo Implantation in Mice

**Figure 5:**
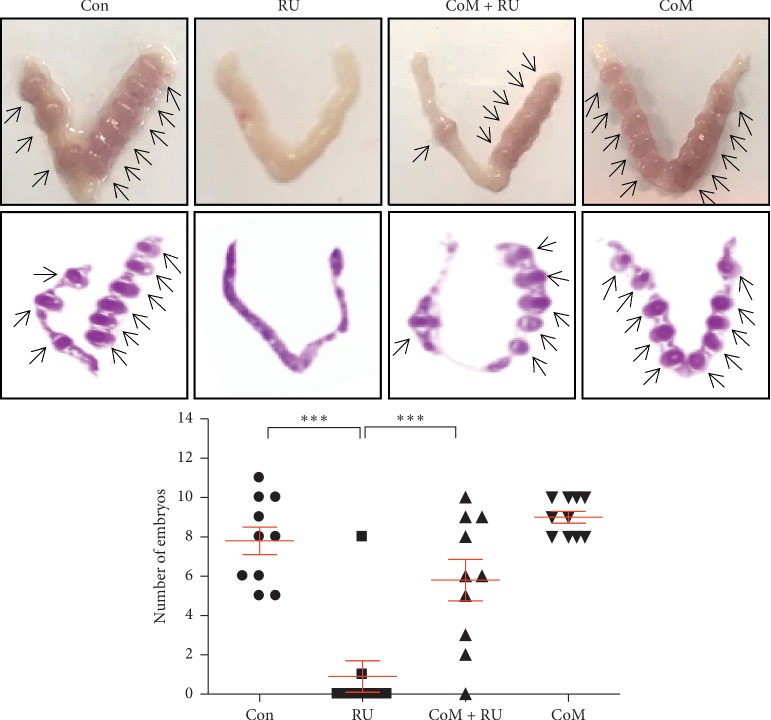
In vivo effect of CoM administration on embryo implantation. To confirm improvement of embryo implantation by CoM, female mice were orally administered with CoM (67.75 mg/kg/day) in 100 *μ*L of normal saline solution for 15 days. On day 4 from pregnancy estimated by vaginal plugs, the female mice were injected with RU486 (4 mg/kg/day) for 4 days. All mice were sacrificed, and both uterine horns were excised to examine the number of implanted embryos. The number of implantation sites was calculated as means ± SEM. Uterine tissue sections were analyzed histologically after hematoxylin and eosin staining. ^*∗∗*^*p* < 0.01 and ^*∗∗∗*^*p* < 0.0001 for comparisons between two groups.

To determine the effect of CoM on blastocyst attachment for embryo implantation, we performed CoM administration on RU486-induced implantation failure mouse model in vivo. A simple schematic of the experiment is shown in [Supplementary-material supplementary-material-1]. As shown in Figure 5, the number of implantation sites in the RU group (0.9 ± 2.51) was markedly depleted compared to the Con group (7.8 ± 2.2), whereas CoM + RU group (6 ± 3.33) was significantly higher than the RU (9 ± 0.94) group.

## 4. Discussion

In recent years, many research studies have focused on the control of endometrial receptivity [20]. Even in traditional medicine in Eastern Asia, Zhong et al. [21] reported that acupuncture improves endometrial receptivity. The reason is because new approaches are needed for treating female infertility, which has not yet been fully resolved, despite the technological advances on assisted reproductive technologies [22]. Thus, our research has explored from traditional herbs to improve endometrial receptivity as another option of infertility treatment. The components of CoM have abundant polyphenol including ferulic acid, caffeic acid, cnidilide, senkyunolide, and ligustilide [23]. These polyphenol components of CoM have pharmacological properties as an anticancer agent through induction of cell death by the expression of Bax/p53 gene and antiangiogenic activity through inhibition of retinal neovascularization [9, 10]. It also has anti-inflammatory activity in LPS-induced murine macrophages via suppression of JNK, ERK, and STAT signaling pathways [24]. However, several major components, such as ferulic acid and caffeic acid, were not effective to improve the endometrial receptivity nor LIF expression (data not shown). To elucidate which compounds of CoM are effective in endometrial receptivity, further extensive experiments are needed.

Many researchers have shown that diverse molecules, such as LIF, interleukin-1 (IL-1), and colony-stimulating factor-1 (CSF-1), play an important role in endometrial receptivity for blastocyst implantation [25–27]. Among them, LIF is the main cytokine regulating the endometrial receptivity [28]. In addition, the embryo implantation process for successful pregnancy involves the adhesion of trophoblast to the receptive endometrium [29, 30]. Significant changes in the expression of adhesion molecules in endometrium have effects on endometrial receptivity. Thomas et al. [31] have reported the increased expression of integrin *α*V*β*3, as an important marker of endometrial receptivity, in the luminal epithelium during the implantation window. Another group has shown that various integrin subunits *α*1, *α*4, *β*1, and *β*5 were known to be expressed in endometrial epithelium during the implantation window [7]. Recently, Kumar et al. [32] showed that integrin *β*8 activates VAV-RAC1 signaling axis via FAK to facilitate the endometrial epithelial cell receptivity for blastocyst implantation. Previously, we also reported that LIF regulates endometrial receptivity via integrins *α*V*β*3 and *α*V*β*5 [18]. The binding of secreted LIF and its receptor in the endometrial epithelium increases expression of integrins *α*V*β*3 and *α*V*β*5 on the plasma membrane surface of endometrial cells, which is involved in the adhesion of the trophoblastic cells to the endometrial cells for blastocyst implantation.

Based on these articles, our results showed that CoM increases the expression of *LIF*, *ITGAV*, *ITGB3*, and *ITGB5* in Ishikawa cells, indicating the promotion of endometrial receptivity for embryo implantation (Figure 3). In addition, the enhanced endometrial receptivity by CoM may affect adhesion of JAr cells to Ishikawa cells (Figure 4). Blocking of LIFR using hLA diminished the adhesion of JAr cells to LIF-stimulated Ishikawa cells which is due to adhesion between JAr cells and CoM-stimulated Ishikawa cell through LIF-dependent pathway. Neutralization of *α*V, *β*3, and *β*5 expression on the endometrial cell surface by using integrin *α*V, *β*3, and *β*5 antibodies significantly reduced the adhesion between trophoblastic cells and CoM-stimulated endometrial cells, suggesting that all integrins *α*V, *β*3, and *β*5 play a critical role in cell-cell adhesion for embryo implantation. Furthermore, these results clearly showed improved implantation of blastocysts in the mouse model of RU-induced implantation failure (Figure 5). Further clinical studies are needed to confirm the effect of CoM on infertile women with decreased endometrial receptivity.

The safety properties of CoM were not validated in this study. Previous study reported that its LD_50_ of i.p. injection in mouse is lower than 34.5 g/kg and LD_50_ of p.o. administration is estimated to 23 g/kg [33]. The dose used in this study (67.75 mg/kg/day) was deduced from a general dose of clinical use of CoM, as previously mentioned in the Material section. In addition, the concentration is much lower compared with a previously reported lethal dose. Thus, we supposed that the dosage might be safe. However, to exactly assess the safety of CoM, further acute and chronic toxicological studies at good laboratory practice level should be performed.

**Figure 6:**
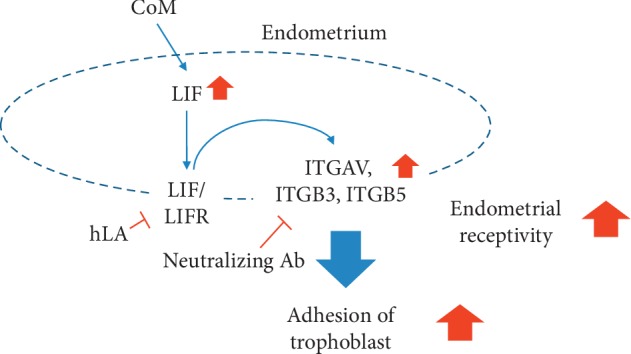
Schematics briefly describing adhesion of trophoblast to endometrium by CoM. This study provides evidence that CoM increased adhesion of trophoblast to endometrium through the enhanced expression of LIF, integrin *α*V, *β*3, and *β*5, indicating improved endometrial receptivity.

In conclusion, as illustrated in Figure 6, CoM enhances the expression of *LIF*. The expression of *ITGAV*, *ITGB3*, and *ITGB5* in CoM-treated endometrial cells also increases the adhesion of trophoblastic cells to endometrial cells for blastocyst implantation. Blocking of LIFR using hLA and neutralization of integrin subunits *α*V, *β*3, and *β*5 using antibodies efficiently inhibits the adhesion of the trophoblastic cells to the CoM-stimulated endometrial cells. Therefore, our results suggest that CoM has a therapeutic potential for ameliorating female infertility related to reduced endometrial receptivity.

## Figures and Tables

**Table 1:** Condition of mobile phase for chromatographic separation.

Time (min)	Solvent A (%)^a^	Solvent B (%)^b^
0	100	
30		100
35		100
36	100	0
43	100	0

^a^Distilled water, ^b^0.1% (v/v) trifluoroacetic acid in water.

## Data Availability

The data used to support the findings of this study are available from the corresponding author upon request.

## Supplementary Materials

Supplementary Materials Figure S1: schematics representation of in vivo experiment process.
