# Supplementary figures and images for: Enhancement of Endometrial Receptivity by Cnidium officinale through Expressing LIF and Integrins
Source: Evid Based Complement Alternat Med. 2019 Nov 16;2019:7560631. doi: 10.1155/2019/7560631 (PMC6885254; doi:10.1155/2019/7560631)

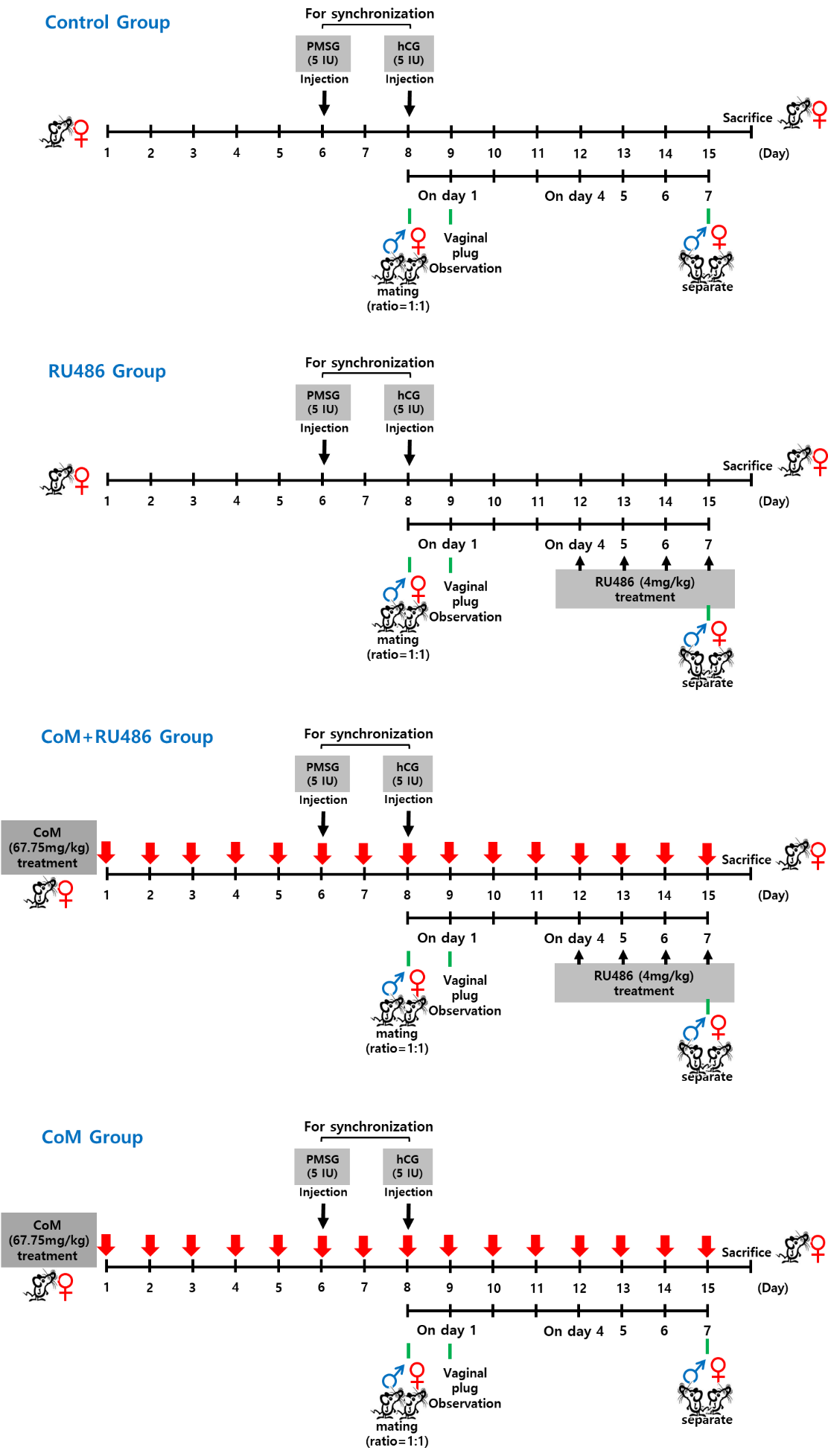


**Fig. S1. Schematics representation of in vivo experiments process.**

Supplement: Supplementary Materials — Figure S1: schematics representation of in vivo experiment process. [file 7560631.f1.docx]
